# Hints and Improvements in the Practice of Medicine and Surgery

**Published:** 1799-09

**Authors:** 


HINTS AND IMPROVEMENTS

IN THE PRACTICE OF

### MEDICINE AND SURGERY.

### *Remarks on the Cure of Spasmodic Asthma.*

DR. UNZER, in his valuable Manual of Medicine, page 1256, and foll has given some hints which well deserve to be communicated to our readers. According to this writer, spasmodic asthma generally requires bleeding, where obvious plethora or hemorrhoidal affections, are the pre-disposing cause of the disease; or when obstinate spasms warrant the application of extreme remedies: although venesection has been found sometimes rather to promote than to prevent the fatal catastrophe.

If the danger of suffocation does not require immediate relief, we may frequently and successfully apply a good number of leeches, which may be put to the breast, the back, or whatever part seems to indicate the use of them, and particularly to the hemorrhoidal veins, where Dr. Unzer has in several instances observed them to afford singular relief. At the same time, two or three pretty strong injections ought to be given at one hour's interval from each other, and after these, opium may be safely administered. Thus, for instance, he gave thirty drops of laudanum; or a mixture consisting of an ounce and a half of peppermint water, five and twenty drops of laudanum, the same quantity of the oleum C. C. and two drams of common syrup, to be taken at one draught.

If, however, the spasms should be connected with an acrimony of the fluids, in such cases the squill, with opium and salt of hartshorn, may be given with advantage. This method may be adopted in all instances where speedy relief is required, whether the breast be idiophatically affected, or by sympathy with the stomach.

A very obstinate spasmodic asthma was, according to Dr. WEIKARD, at length removed, by rubbing the feet every night with the tincture of cantharides.

Another German physician, Dr. LENTIN, asserts, that four grains of musk have proved beneficial in an inveterate case of spasmodic asthma; and that, in general, the anti-hysteric medicines may be safely and often successfully prescribed in every species of asthma. Sometimes indurated glands, particularly in the neck, are the collateral symptoms of spasmodic asthma, and in such cases, the anti-hysteric remedies deserve the principal attention of the practitioner, while the spasmodic paroxysms ought to be treated like nervous affections arising from spasms.

LE COMTE, a French writer, relates the following extraordinary case of a woman, who was in other respects healthy, but had from her infancy, almost every night, been troubled with an asthmatic paroxysm, increasing with her years. On account of indurations in the breasts, this patient was directed to take the extract of cicuta internally, which not only discussed the callosity of her breasts, but likewise alleviated her asthmatic complaint, and at length completely cured it. She took this medicine during the almost incredible time of four years, in the evening, in increased doses, so that she at length usually swallowed forty grains for a dose; nay, she at one time increased the doſes to one hundred and twenty, and one hundred and forty-ſix grains; which, however, produced a violent narcotic effect, inſomuch that when ſhe recovered from this ſtupefaction, all objects appeared to her remarkably diminiſhed. Sometimes ſhe would ſuſpend the uſe of the medicine for eight or nine days; and during the term of about two years and a half ſhe had conſumed no leſs than nineteen thouſand grains of the extract of hemlock.

If external ſtimuli, ariſing from acid vapours, ſhould occaſion a ſpaſmodic aſthma, it will be readily perceived, that the only remedy is the removal of the exciting cauſe.—Thus, in the vicinity of Mount Veſuvius, the inhabitants were attacked with a convulſive aſthma, after an eruption of this volcano, and moſt of the patients died on the third day, obviouſly from the fatal effects of veneſection.

An ingenious Italian phyſician, VIVENZIO, on the contrary, preſcribed mucilaginous remedies, and the ſteam of a decoction of emollient herbs; and as ſoon as the ſenſe of ſuffocation was overcome, he ordered twelve ounces of blood to be drawn, and the inhalation of emollient vapours to be continued. In this manner, ſays DELLA TORRE, Vivenzio ſaved the life of almoſt every patient, without an expectoration or any remarkable criſis having taken place.

[*To be continued in our next Number.*]

### *A ſingular Caſe of Anaſarca of the Liver.*

DR. SACHSE, a German practitioner at Uelzen, relates, in the tenth Number of the "*Journal der Erfindungen,*" the following remarkable caſe, which we are inclined to term an *abſceſs*, rather than *dropſy* of the liver.

A farmer's wife, thirty years of age, applied to him on the 4th of March, 1794, complaining of a hard, thick, and painful ſwelling, which extended from the ſhort ribs of the right ſide to the pit of the ſtomach, and ſeemed to occupy the ſeat of the large right lobe of the liver.—The colour of her eyes as well as the ſkin, was natural; reſpiration ſomewhat difficult; the pulſe ſmall and rather irritated; the heat of the body natural; the *fæces* of a proper conſiſtence and colour; decumbence on the right ſide difficult; ſleep tolerably eaſy; the menſes regular; no ſweats at night, no ſhiverings accompanied with heat, nor apparent emaciation.—Formerly ſhe had enjoyed good health, excepting ſome pains in her back and ſhoulder. Since the laſt 12 months ſhe could not bear her ſtays tightly laced; and about three months ago ſhe was ſeized with a burning pain in her right ſide and ſhoulder: ſhe loſt her appetite, which had uſually been good; was troubled with nauſea aud frequent vomiting, accompanied with febrile ſymptoms, inſomuch that ſhe was for five days confined to her bed. At that period the ſwelling in the right ſide appeared, which, however, was diſregarded, while all other ſymptoms yielded to ſimple family remedies, although ſhe felt, without intermiſſion, a ſenſe of preſſure and burning heat in the part affected. Theſe ſenſations, about three weeks previous to her conſulting him, had become more intenſe, and the ſwelling of the part more extended.

The circumſtances of the caſe here detailed, induced Dr. Sachſe to believe that three months ago ſhe had been afflicted with an acute inflammation of the liver, which had ſince been changed into a chronic induration. Hence he preſcribed the taraxacum, ſenega, and radix graminis, in decoctions; antimonial remedies with gentle cathartics; and directed mercurial ointments to be rubbed in, with the neceſſary precaution. A week after theſe preſcriptions, ſhe again applied to Dr. Sachſe, who reſided at ſome diſtance from the patient's abode, and complained that ſhe had experienced no relief; that on on the contrary, her pains had become more violent, the fwelling more extenfive, and that fhe was troubled with flight fhiverings and frequent flying heat. Thefe fymptoms, together with an obfcure fluctuation he perceived in the part affected, which could not well bear to be touched, made him fuppofe that there was a collection of purulent matter, and that it was an abfcefs of the liver, which required to be opened by the lancet. Yet he was too timorous to venture on the operation, and directed therefore emollient poultices, pointing out the fpot to which a roafted onion fhould be applied: this he did with a view to effect a more certain concretion of the liver with the peritoneum. In two days he faw her again; fhe could not fupport the very excruciating pain but in a reclined pofture; the fluctuation appeared ftill more diftinct, and the operation was now determined upon. The incifion was made to the depth of an inch, and there appeared no *pus*, but, contrary to all expectation, an effufion of *water* took place, which inftantaneoufly alleviated the pains of the patient. The water flowed without intermiffion till about four pounds and a half had been evacuated; it iffued more freely when the patient bent herfelf backwards; was at firft of a yellowifh, afterwards a reddifh, and then of a completely white colour; had a faline and fomewhat fweetifh tafte; coagulated quickly, and at length had the appearance of weak hartfhorn jelly: on ftirring it, tough yellowifh threads feparated from it, which extended above the length of a yard without breaking, fimilar to thofe formed by the blood when agitated with rods. While the water, or rather lymph was ftill trickling out of the fmall wound, he applied a pledget and covered the orifice with thick compreffes, to exclude the accefs of air: thus it became gradually fmaller, and healed without difficulty in eight days. Internally, Dr. Sachfe directed the ufe of antimonial remedies, with bark and fmall dofes of rhubuarb, which, after having been continued for feveral weeks, effected a cure, excepting that the patient ftill feels a continued but inconfiderable induration of the liver. And although fhe has remained in good health for a twelvemonth after this attack, yet Dr. Sachfe remarks, that time only can determine whether fhe will undergo a relapfe.

---

### *An Account of the Cachexia Africana.*

As we doubt not, that this article will be found particularly worthy the attention of our readers, we have extracted it from the "*Medical Repofitory*" printed at New-York, conducted by Drs. MITCHELL and MILLER. The author of it is Dr. CHISHOLM.

The fimilarity between this difeafe, to which the negro flaves lately imported into the Weft-Indies are fubject, and one to which young females are liable, in moft countries, we prefume will not efcape the obfervation of practical readers: and we do not hefitate to pronounce, that the fame theory may be fafely applied to both.

"There is," fays Dr. Chifholm, "a difeafe to which the negroes, and particularly thofe lately imported are much fubject: it is named by us *Mal d'Eftomac*, or *Cachexia Africana*; and from a conftant fymptom which attends it, *dirt-eating*, by fome. (Dr. HUNTER; Difeafes of Jamaica.) Negroes alfo, who have been fome time in the country, are fubject to this difeafe, but not fo frequently as amongft the former. It affects thofe who have, generally fpeaking, been badly clothed, ill-fed and lodged, and whofe conftitutions have been worn out by hard labour. The mind partaking of the fufferings of the body, is affected with noftaglia, brooding over their ill-treatment, feparated from friends and relations, and doomed to fuffer without daring to complain.

"The

"The firſt ſymptom, and which indeed is both the cauſe and effect, is a fondneſs for ſolitude, ſadneſs, grief, and deſpondency; a loſs of appetite, or a deſire only for what is pungent and ſtimulant; difficulty of breathing, eſpecially in walking up a hill; a painful gaſtrodynia; palpitation of the heart; general debility; drowſineſs; paleneſs of the face and palms of the hands; the tongue white, ſometimes with an appearance like ſtains of ink upon it; the lips colourleſs; the tunica adnata of a glaſſy whiteneſs, as alſo the teeth; the ſkin of an olive complexion, and cold to the touch, with a rough ſurface, and the papillæ elevated; anaſarcous ſwellings of the eye-lids, face, and extremities; water is afterwards collected in the belly and cheſt, and the unhappy ſufferer can only breathe in an erect poſture, for fear of inſtant ſuffocation; the pulſe is always ſmall, and generally becomes quicker towards night. There is, during the diſeaſe, an unwillingneſs to attempt, and inability to perform motion.

"Morbid changes take place throughout the alimentary canal, in conſequence of the vitiated ſtate of the gaſtric juice and impeded digeſtion; a morbid acidity prevails, and a ſymptom ariſes from this cauſe, which, with ſome has given name to the diſeaſe,—a habit of eating chalk, dirt, or whatever will obtund acrimony.

"This vitiated action is propagated throughout the whole alimentary canal; the lacteals are abraded by acrimonious fluids, and no longer poſſeſs the power of abſorbing healthy chyle; hence the lymphatic glands of the meſentery become inflamed and indurated. The blood poor, vapid, and colourleſs, no longer ſtimulates the heart and arteries to action; hence aſphyxia and ſudden death, and thoſe polypous concretions found in the heart after death.

"It is to the want of irritability of the blood that we are to aſcribe obſtruction of the menſtrual flux in women, in this diſeaſe.

"From this ſhort account of the diſeaſe, which has no other merit than truth, you will be prepared for the appearances upon diſſection:

"The ſtomach is found much enlarged, and thickened in its coats; the liver ſometimes enlarged and ſchirrous, but always preternaturally white; the gall-bladder ſometimes with biliary concretions; the bile never of a healthy appearance, generally thin and watery, and ſlightly yellow or green: the meſenteric glands indurated and ſchirrous. Thoſe appearances induced a medical practitioner in a neighbouring iſland to employ mercury, with a view of removing, as he ſuppoſed, obſtructions, but a very ſmall quantity of it excited ſuch terrible effects as obliged him to deſiſt. Accumulated irritability, from the abſtraction of the uſual ſtimuli, had rendered them more ſuſceptible of the ſlighteſt ſtimulus.

"From this view of the diſeaſe, the pathology and treatment of it, I conceive, will be eaſily underſtood Reſembling ſcurvy, in ſome reſpects, it differs from it only in the ſymptoms of putrid diatheſis not being ſo obvious—putrid animal food not having been here employed as an article of diet. The ſame defect of oxygen prevails in both diſeaſes; and it is probable, that we would find the ſame benefit from the uſe of aceſcent vegetables; but we have not here the ſame putrid diatheſis to obviate; and the ſtomach has been already ſo much debilitated by weak vegetable diet, that it requires a more ſtimulant plan—animal food, wine, warm clothing, a gentle treatment, &c. The preparations of iron are here found of the moſt eſſential ſervice. Much benefit has alſo been derived from weak fermented liquors—aceſcent cane-liquor has cured many.

"Perhaps there may be alſo a deficiency of carbon. I am inclined to think that the lungs have a greater ſhare in ſeparating oxygen than the ſtomach ftomach and alimentary canal; but from the mutual fympathy which fubfifts between the ftomach and lungs, the vigorous exertion of the former is required to enable the lungs to perform their peculiar action. I conceive, that to produce fcurvy, the abftraction of the ufual ftimuli, as in the difeafe, the hiftory of which I have juft now given, will be fufficient.

"It is remarkable that negroes, fubject to this difeafe, have been much benefited by living in a low fituation, near marfhes, which quickly prove fatal to whites; and I have long obferved this, before I had formed any theory upon the fubject. Perhaps the hydro-carbonic air may act as a cordial—it is perhaps the nervous ether itfelf. It has been remarked by medical writers, that the attack of remittent marfh fevers is frequently preceded by an unufual flow of fpirits."

---

### *A Cafe of Epilepfy, treated by Argentum Nitratum.*

The following cafe has been communicated to the Editors of the Medical Repofitory, by Dr. J. E. White, of Waynefborough, in Georgia; and as the fubject of epilepfy has already engaged the attention of our readers, in the fecond and fixth Numbers of this work, we do not hefitate to infert it.

"The patient," fays Dr. White, "was a boy aged fix years. He had laboured under the difeafe from the time he was eighteen months old. The fits occurred moft frequently in the night, generally to the number of four or five; but he has been known to have fixteen in that fpace of time. He rarely paffed a night without having more or lefs, except once, for about two months, when they entirely ceafed; but they feldom recurred during the day. Various remedies had unfuccefsfully been tried, and the fits had now become worfe than ever they had been known, returning very frequently, both night and day.

"I was applied to, and thought it a fair cafe for giving the nitrated filver: I accordingly directed it in the following manner:

℞ Argent. nitrat.
Medull. pan. aa. fem. drachm. and divide into thirty pills; one to be taken night and morning.

"After he had taken four of the pills, the dofe was increafed to three in the day, (one being taken at noon) without producing any fenfible effect. He had continued their ufe but four days, before the fits feemed to be fuddenly arrefted in their progrefs towards a fatal termination, and he remained entirely free from them for nearly two weeks. He had now a very flight return of them, and I ordered five pills to be given in the day. This dofe was continued for a fhort time, when I increafed the quantity of the argentum nitratum in each pill, to one grain and a half, of which he took four in the twenty-four hours, for a few days, (ftill remaining entirely clear of his complaint) when he was feized with the fever of this climate, which, from inattention, quickly put a period to his exiftence.

"Though it cannot be determined what would have been the iffue of this cafe, yet, in my opinion, the moft unequivocal proofs were given of the good effects of the medicine, and I fhall not fail, in future, to make trial of it in cafes that I may deem proper for its exhibition."

---

### *Obfervations on the Nature and Cure of Hydrophobia.*

The Medical works of Dr. Rush, of Philadelphia, are too well known and efteemed, both in Europe and America, to require any additional commendation. This original writer has lately publifhed the fifth volume of his

"*Medical Inquiries and Observations*," from which we have selected the following article: and as we find a concise and satisfactory view of it given by the Editors of the "*Medical Repository*," in their fourth Number of the second volume, we do not hesitate to avail ourselves of their well-executed labours.

The exertions of physicians to lessen the number of incurable diseases form a splendid part of the modern history of medicine. Success has not always crowned the endeavours directed towards this object; but no effort has been wholly lost; and the improvements made, in many instances, have been such as might be sufficient to incite even the most tardy in this career of usefulness.

The terrors of hydrophobia have long held possession of the minds of men. Few instances of the successful treatment of it are to be found upon record, and even these few are supposed by many to be of dubious authority. Our learned author, therefore, has not declined any difficulty in selecting the subject of these observations.

Dr. Rush assigns the following as some of the remote and exciting causes of hydrophobia: the bite of a rabid animal—cold night air—a wound in a tendinous part—putrid and impure animal food—worms, &c. And the theory of the disease, which an examination of its causes, symptoms, and accidental cures, has led him to adopt, is, that it is a *malignant state of fever*. He is induced to think so from the febrile nature of the disease in all rabid animals—from its prevalence as an epizootic, at the same time that malignant fevers are epidemic—from the resemblance between the symptoms of canine madness and malignant fevers—and from the appearances of the bodies of dogs dead of the disease, as discovered by dissection.

Dr. Rush concludes, that the disease produced in the human body, by the bite of a rabid animal, is of the nature of a malignant fever—from its symptoms—from its appearing, like a malignant fever, at different intervals after the time of receiving the infection—from the similiarity of the appearances of the blood, when drawn in both cases—from the agreement of the diseases in point of duration—from the equally rapid putrefaction of bodies dead of either disease—and from the sameness of appearances in the dead bodies upon dissection.

The remedies for hydrophobia are divided by our author into two kinds: 1. Such as are proper to prevent the disease, after the infection of the rabid animal is received into the body. 2. Such as are proper to cure it when formed. Under the former head of remedies, he mentions cutting or burning out the wounded part—long and frequent affusion of water—and keeping the wound open and running for several months. He recommends low diet as a means of prevention, and supporting the spirits and confidence of the patient; but he does not rely upon mercury for this purpose, as the disease has been known to come on, notwithstanding a salivation.

As soon as the disease is discovered, Dr. Rush urges the use of blood-letting as the principal remedy, and advises that the quantity drawn be very copious. Many instances are adduced of the bold employment of this remedy with success. Besides blood-letting he recommends purges and clysters, sweating, and salivation by mercury. He also mentions cases where musk and opium, bark and wine, have been found efficacious remedies. Blisters and stimulating cataplasms, he supposes, may be useful in the decline of this disease, as they are in that of malignant fevers. The cold bath, also long immersion in cold water, have produced beneficial effects.

The analogy between hydrophobia and malignant fevers, which is so ably supported by Dr. Rush in these observations, seems to be founded upon a just a just survey of the action of poisons upon the animal system. However various the substances called poisons may be, in respect either of their constitution or the effects they may occasionally produce, we are well persuaded they all hold many principles in common, and that a multitude of them attack animal life in a mode essentially the same.

---

### *On the Use of the Digitalis in Dropsy, Consumption, &c.* *

From a letter addressed to us by Dr. SHERWEN of Enfield, Middlesex, dated August 8, 1799, we extract the following particulars:

"The favourable report which has been given of the effect of the *Digitalis Purpurea*, in the *Cure of Consumption*, has already excited, and will doubtless, still more excite, the attention of the faculty. I wish it may not also induce the public at large to make too free with that poisonous plant.

"The good effects of digitalis in the cure of dropsy, are now well known; and I believe there are few medical men ignorant of the fatal effects which it is also capable of producing, and which it frequently has produced, even when administered under the direction of very skilful practitioners. It is a duty therefore incumbent upon those who are endeavouring so meritoriously to *revive* the reputation of digitalis, to unite their encomiums with proper cautions respecting those insidious and deleterious properties of this plant, which have doubtless been the reason why it fell into disuse during the greatest part of the present century.

"That there is nothing new under the sun, is an old adage, not badly exemplified upon the present occasion; for the following encomiums upon the anti-phthisical virtues of digitalis may be read in "SALMON's *Botanologia*, or *British Herbal*," a large folio volume, published about one hundred years since.

"It is a specific which transcends all other vegetable medicaments for the "cure of consumptions; cleansing and healing after an admirable manner "ulcers of the lungs. It opens the obstructions of all the viscera, cleanses, "carries off, or expels the recrements of the humours, by which means "the daily nutriment may be conveyed to all the parts of the body. "The syrup or rob of the juice of the herb and flowers, made with honey, "may be given morning and night, four or five spoonsful at a time, accord-"ing to age or strength of the patient. Some advise three spoonsful to be "taken in mead, in the morning fasting, as much at ten in the morning, "three spoonsful at four in the afternoon, and lastly, as much going to bed. "This medicament has restored (where the patient has not been absolutely "past cure), beyond all expectation. It cures a phthisick or ulceration of "the lungs, when all other medicines have failed, and the sick been "esteemed past cure. It opens the breast and lungs, frees them from "tough phlegm, cleanses the ulcer, and heals it, where all other remedies "act without effect. I have known it do wonders, and speak here from "a long experience. Persons in deep consumptions, and given over by all "physicians, have, by the use of this syrup, or rob, been strangely reco-"vered, and so perfectly restored as to grow fat again. I commend it as a "secret, and it ought to be kept as a treasure. I am very confident of it; "the deplorable wasted patients who have been long languishing in an inve-

"terate

---

* On this subject we have already inserted, in a former part of this Number, p. 115 and foll. an interesting paper communicated to us by Dr. MACLEAN, to which we beg leave to refer our readers.

" terate and tedious consumption, or a phthisis, if they make use hereof, " will give me thanks for this notice, whilst they may have reason enough " to curse even the memories of quacking blood-suckers, issue makers, and " blister-drawers, who, as they may have possibly drained them of a fair " part of their estate and treasures, would, by a continuance under their " hands (for all their specious methods of cure) have fooled them out of " their lives too. But here it is to be noted, that this syrup ought chiefly " or only to be made of the flowers." *

" Very little is mentioned by Salmon of its virtue in the cure of dropsy: he says indeed, in a general way, that it is " *abstersive, emetic, cathartic,*" that it " cleanses and purges the body both upwards and downwards, freeing it both from viscous and *watery* humours." He also adds a caution, " that it ought not to be given in too great quantity, because of its *violent* operation;" but he appears to be a perfect stranger to all the nervous distress and deadly influence over the vital principle which it sometimes produces, entirely independent of its evacuating powers. Although he was unacquainted with this part of its character, he speaks positively of its good effects in the cure of epilepsy, " and by late experience" (he says) " it " has been found effectual against the falling sickness, for that divers have " been absolutely cured thereby."

" When, to the popular encomiums of Salmon at the commencement, we add those of the late Doctor Alston, of Edinburgh, about the middle of the present century, it is I think fair to conclude, that an herb, the virtues of which were so generally known, could not have fallen into disuse but for good reasons: and although these have not been assigned by any of the older medical authors, from what we now know of its deleterious nature, there can be no doubt but numbers must have been injured by it; and I hink we have every reason to expect that the same will be the case again; as the public are ready to catch eagerly at every new remedy for consumption; particularly when recommended by men deservedly high in their esteem.

" The above, together with the modern encomiums, afford satisfactory evidence, that the digitalis is a valuable remedy; and since we are now well acquainted with its poisonous properties, there can be no doubt but in the hands of prudent and attentive practitioners, it will be a valuable addition to the modern Materia Medica, in the treatment of consumption, as well as dropsy; and it must be added, that the assertion of Salmon (although he was never an author of merit or reputation †) respecting its salutary influence on *epilepsy*, deserves the most serious attention ‡. The fashion

* Obsolete as Salmon's Botanologia is, this extract from it was submitted to my opinion by a lady who was preparing it for her friend in the last stage of consumption: I am persuaded that the modern encomiums of digitalis were not known to the friend who sent it her. I. S.

† A pleasant anecdote is told of an Auctioneer who had the sale of some books, one of which, a work of Salmon's, had been in the possession of Dr. Radcliffe. This the auctioneer puffed most violently, as a work of the *ingenious* and learned Dr. William Salmon, with marginal manuscript Annotations by Dr. Radcliffe. This excited attention, as the great Doctor Ratcliffe had never been known to have *written* any medical observations, and the book sold at a high price. When the purchaser came to examine his treasure, the hand-writing was certainly that of the Doctor, and to the following effect: ' This is the most cursed stupid book that ever was published." —" ignorant blockhead"—" booby"—" jack-ass" &c. &c.

‡ Admitting the facts, which perhaps there can be no good reason to doubt, may not the good effect of the digitalis in this instance also depend on its property of exciting absorption or promoting a free discharge of urine. There is some reason to believe that epilepsy may often depend on an over-proportion of water or lymph in the ventricles of the brain, without its amounting to actual hydrocephalus.

faſhion of the day, and the high authority of the College * tend ſo much to encourage the uſe of poiſons, that there can be no doubt but the digitalis will be very generally preſcribed by profeſſional men, and I ſincerely wiſh its adminiſtration may remain in their hands.

"The theory of retarding the circulation for weeks together, has a very promiſing ſound, reſpecting ſome of the ſymptoms and circumſtances connected with phthiſis pulmonalis; but the digitalis could never have been preſcribed with that view by Salmon and his contemporaries. Perhaps the ſalutary effects of this medicine in conſumptive caſes may, in ſome inſtances, be referred entirely to its diuretic property: ſerous effuſion into the cavity of the thorax is a very common conſequence of active inflammation in the lungs; emaciation, cough, night-ſweats, hectic fever, and copious expectoration of mucus of a purulent appearance, may, and often do, follow inflammation in the lungs, where neither abſceſs nor ulceration has taken place; and it is eaſy to conceive that all theſe ſymptoms may be combined with hydrops pectoris; and that a patient, gradually ſinking under their combined influence, may have been ſpeedily reſtored to health, by the adminiſtration of a medicine capable of producing ſuch happy effects as we now frequently witneſs in the moſt dangerous ſymptom, the hydropic oppreſſion; that being removed, all the other appearances of phthiſis pulmonalis would gradually ſubſide, and the digitalis acquire the reputation of curing a conſumption which had never exiſted.

"I am afraid that a greater number of facts, and more experience muſt be brought forward, before we can adminiſter it with much confidence in the true ſcrophulous conſumption. It is, however, worthy of remark, that Salmon in his ſpecification of its virtues ſays, "it cures conſumption, *king's* "*evil*, green ſickneſs, and falling ſickneſs, alſo wounds, old ſores, and running ulcers." Hence it is evident, that he conſidered it as uſeful in ſcrophula; and we have reaſon to ſuppoſe that his obſervations and encomiums are the reſult of *experience*; becauſe he appears to have known very little of its virtues, when he wrote his "*New London Diſpenſatory*, with Remarks," the imprimatur of which bears date thirty years antecedent to the "Botanologia."

* Nostris temporibus alia est, et longe dissimilis *venenorum* fortuna; neque enim ab iis, tanquam prorsus inimicis, abhorrere videtur medicina, sed ea ad partes suas traducere, et opem eorum sociam et adjutricem exposcere."

*Pharmacop. Coll. Reg. Medic. Lond.*

